# Weekly bacterial community profiles spanning the 2023 Carnivale season sourced from two wastewater treatment plants in southeastern Louisiana

**DOI:** 10.1128/mra.01330-25

**Published:** 2026-03-24

**Authors:** Joe Berta, Lori Rowe, Bob Garry

**Affiliations:** 1 U.S. Naval Research Laboratory, Stennis Space Center, Mississippi, USA; 2 Tulane University VCIPS Core, Tulane National Primate Research Center 5783, Covington, Louisiana, USA; 3 Microbiology Department, Tulane University School of Medicine 12255, New Orleans, Louisiana, USA; University of Southern California, Los Angeles, California, USA

**Keywords:** wastewater-based epidemiology, mass gatherings, 16S metabarcoding

## Abstract

To understand changes in New Orleans’ wastewater microbial community resulting from tourism surges surrounding Carnivale, we characterized the bacterial diversity present in municipal wastewater from New Orleans East Bank and Mandeville Public Works wastewater treatment plants using 16S rRNA metabarcoding. Samples were taken every Thursday for 10 weeks, spanning the 2023 Carnivale season.

## ANNOUNCEMENT

Human mass gatherings can have a substantial impact on the bacteria circulating in their human host populations (1). These bacteria then typically make their way into the local municipal wastewater, where the species richness and abundance of an entire human population can be analyzed (2).

To study this phenomenon during the 2023 New Orleans’ Mardi Gras celebration, we collected 24-h composite, influent samples from the New Orleans, LA, Eastbank wastewater treatment plant (WWTP) every Thursday starting on 12 January 2023 and ending on 16 March 2023. To serve as a control that experiences similar weather (ambient temperature and precipitation) but negligible tourism over the same time frame, matched samples were also collected from the Mandeville Public Works WWTP. All samples were 1 L in volume when collected via an autosampler. The New Orleans Eastbank WWTP has a catchment of around 330,000 people, while the Mandeville WWTP has an approximate catchment of 13,000.

The samples collected are described in Table 1. Samples were stored at −80°C until processing. Samples were homogenized for 10 min. Three hundred fifty milliliters of each 1 L sample was aliquoted for testing. We removed large solids from the samples through pelleting via centrifugation (500 × *g* for 5 min). The prokaryotic fraction was collected from the supernatant using high-speed centrifugation (RCF_AVE_ of 12,000 × *g* for 15 min). We extracted prokaryotic DNA using Zymo’s Quick-DNA/RNA Miniprep Plus Kit with the addition of their 2 mm BashingBead Lysis Tubes, which were used for 30 min. Nucleic acid recovery was measured using NanoDrop OneC. Library prep was conducted in accordance with Illumina’s 16S Metagenomic Sequencing Library Preparation protocol (3). Briefly, two rounds of PCR were conducted, one for the target amplicons and one to add indices and sequence adapters. Both PCR sets involve minimal rounds of PCR to limit sequence bias introduced by PCR amplification (25 and 8 rounds, respectively).

**TABLE 1:** Description of samples taken from two southeastern Louisiana wastewater treatment plants: New Orleans and Mandeville, LA^*a*^

Sample	WWTP	Date (day-mo-yr)	Sample sequencing run name	Extraction yield (ng/mL)	A260/A280	Raw reads	Trimmed reads (thousands)	Richness (species OTUs)	Shannon entropy (bits)	SRA accession no.
Week 1	NOLA	12-Jan-23	A1	8.4	1.65	591,816	588	242	3.89	SRX31058772
Week 2	NOLA	19-Jan-23	A2	12.7	1.55	734,882	729	253	4.29	SRX31058773
Week 3	NOLA	26-Jan-23	A3	18.0	1.59	646,064	641	253	4.14	SRX31058784
Week 4	NOLA	02-Feb-23	A15	5.8	1.84	613,192	609	287	4.21	SRX31058785
Week 5	NOLA	09-Feb-23	A5	6.9	1.84	653,489	609	211	3.95	SRX31058786
Week 6	NOLA	16-Feb-23	A11	7.6	1.31	592,774	589	206	4.34	SRX31058787
Week 7	NOLA	23-Feb-23	A12	3.0	1.63	747,072	742	218	4.19	SRX31058788
Week 8	NOLA	02-Mar-23	A14	6.7	1.38	838,717	833	264	4.42	SRX31058789
Week 9	NOLA	09-Mar-23	A4	7.2	1.19	736,308	731	231	4.30	SRX31058790
Week 10	NOLA	16-Mar-23	A13	11.4	2.16	755,778	750	211	4.28	SRX31058791
Week 1	Mandeville	12-Jan-23	A1-2	0.02	3.37	137,497	135	85	3.76	SRX31058774
Week 2	Mandeville	19-Jan-23	A2-2	0.04	2.46	117,874	115	98	3.79	SRX31058775
Week 3	Mandeville	26-Jan-23	A3-2	0.03	2.10	134,998	132	100	3.74	SRX31058776
Week 4	Mandeville	02-Feb-23	A4-2	0.05	2.11	96,229	94	86	3.79	SRX31058777
Week 5	Mandeville	09-Feb-23	A5-2	0.04	2.30	118,822	117	103	3.92	SRX31058778
Week 6	Mandeville	16-Feb-23	A6-2	0.03	3.06	94,299	93	71	3.87	SRX31058779
Week 7	Mandeville	23-Feb-23	A7-2	0.05	1.57	119,211	117	89	3.97	SRX31058780
Week 8	Mandeville	02-Mar-23	A8-2	0.04	1.99	118,820	117	85	3.82	SRX31058781
Week 9	Mandeville	09-Mar-23	A9-2	0.08	2.11	116,704	115	80	3.53	SRX31058782
Week 10	Mandeville	16-Mar-23	A10-2	0.06	2.01	94,258	93	68	3.86	SRX31058783

^*a*^ Richness was calculated as the total number of unique, species-level operational taxonomic units observed in the sample. Shannon entropy was calculated using the Shannon-Wiener Index, which is the negative sum total of the product of each species’ relative abundance multiplied by the natural logarithm of that same relative abundance.

The circa 400–460 bp amplicons from the V3–V4 regions of the bacterial 16S rRNA gene were created using the Bakt_341F (5′-CCTACGGGNGGCWGCAG-3′) and Bakt_805R (5′-GACTACHVGGGTATCTAATCC-3′) primers (4). Non-specific forward and reverse overhang adapters bridged the 5′ ends of the gene-specific 341/805 primers to the 3′ ends of P5 and P7 barcode sequences using the Nextera XT DNA Library Preparation Kit (Illumina 15032354). One hundred twenty-five nanograms of each sample was pooled, resulting in a final library pooled concentration of 16.7 ng/µL. The library had a peak height at 630 bp, and the final library was 40.8 nM. This was diluted to 3.82 nM, denatured, and then loaded onto the MiSeq at 10 pM. Cleanup was conducted after each round of PCR using Beckman Coulter’s AMPure XP beads (Fischer NC9933872). Samples were sequenced on an Illumina MiSeq using a v.2 Reagent Kit to generate 300 bp, paired-end reads.

For read analysis, default parameters were used for all programs unless otherwise specified. Sequence quality control was conducted using FastQC (v.0.12.1) (5). Cutadapt (v.4.9) was used to remove adapter sequences, bases with Phred scores below Q20, and reads less than 100 bases in length. Metabarcoding analysis was conducted using Kraken 2 (v.2.1.3) and their 8GB Standard reference index (6). The Kraken 2 confidence threshold was raised to 0.01, and taxa with fewer than 0.004% of the total reads mapped to them were removed. These low-read taxa were determined to be unreliable during benchmark testing. The resulting Kraken reports were then fed into Bracken (v.2.9) for abundance estimation using the 250 bp length parameter (7, 8).

## Data Availability

All raw reads were deposited in GenBank SRA under BioProject number PRJNA1363154; links to individual samples (SRX31058772 to SRX31058791) are in Table 1.
